# Perioperative subconjunctival steroid injection in dropless cataract surgery: a systematic review and meta-analysis

**DOI:** 10.5935/0004-2749.2024-0394

**Published:** 2025-09-10

**Authors:** Laura Goldfarb Cyrino, Dillan Amaral, Alexandre Yamada Fujimura Júnior, Bela J. Parekh, Marcela Marino de Azeredo Bastos, Giovana de Souza Gaio, Maria Antônia Torres Arteche, Amanda Souza do Nascimento, Vitor Expedito Alves Ribeiro, Jaime Guedes, Marianna Almeida Hollaender

**Affiliations:** 1 Universidade de São Paulo, São Paulo, SP, Brazil; 2 Universidade Federal do Rio de Janeiro, Rio de Janeiro, RJ, Brazil; 3 Faculdade de Medicina de Marília, Marília, SP, Brazil; 4 Wills Eye Hospital, Philadelphia, PA, USA; 5 Universidade da Região de Joinville, Joinville, SC, Brazil; 6 Universidade Federal do Rio Grande do Sul, Porto Alegre, RS, Brazil; 7 Universidade Estadual de Londrina, Londrina, PR, Brazil; 8 Universidade Federal do Piauí, Teresina, PI, Brazil; 9 Glaucoma Research Center, Wills Eye Hospital, Philadelphia, PA, USA

**Keywords:** Cataract extraction, Phacoemulsification, Lens implantation, intraocular, Postoperative care, Intravitreal injections, Anti-inflammatory agents, non-steroidal/administration & dosage, Glucocorticoids, Triamcinolone acetonide, Research design, Randomized controlled trial

## Abstract

The advantages and disadvantages of using perioperative subconjunctival steroid injections in dropless cataract surgery continue to be debated. A systematic review of PubMed, EMBASE, and the Cochrane Central database identified five studies—two randomized controlled trials and three non-randomized studies—encompassing 70,751 eyes. Among these, 12,319 eyes (17.4%) received subconjunctival steroid injections, while 58,432 eyes (82.6%) were managed with topical steroids. The Cochrane Collaboration’s RoB 2 tool was applied for bias assessments in randomized controlled trials, and heterogeneity was assessed using the I² statistics. No statistically significant differences were found between the two groups regarding macular edema (p=0.249), visual acuity (p=0.73), or laser flare count (p=0.45). Both subconjunctival injections and topical steroids demonstrated comparable efficacy and safety in controlling postoperative inflammation after cataract surgery. Additional research is warranted to validate these conclusions.

## INTRODUCTION

Cataract surgery ranks among the most frequently performed surgical procedures globally. As reported in the 2020 Vision Report, around 10 million cataract surgeries are carried out each year worldwide, with this number expected to rise due to population growth and increasing life expectancy^(1)^. Over recent decades, notable improvements in the safety of the phacoemulsification technique have led to better refractive outcomes and surgical precision. Despite these advancements, postoperative ocular complications remain a concern. Evidence in the literature suggests that inadequately controlled inflammation is a major contributor to many of these complications, including elevated intraocular pressure (IOP), cystoid macular edema, posterior capsule opacification, posterior synechiae, decentration of the intraocular lens (IOL), epiretinal membrane formation, ciliary membrane development, hypotony, and secondary glaucoma, all of which can lead to discomfort or significant pain for patients^(2)^. These complications may hinder recovery, compromise visual outcomes, and affect overall patient satisfaction^(3)^.

Historically, the standard prophylactic approach to managing postoperative inflammation following cataract surgery has involved a scheduled regimen of topical steroid and/or nonsteroidal anti-inflammatory (NSAID) eye drops administered multiple times daily over several weeks^(4)^. However, this approach depends heavily on proper patient compliance and adherence to medication. One prior study found that 92.6% of cataract surgery patients who self-administered eye drops did so incorrectly, with errors such as not washing hands, contaminating the bottle tip, missing the eye, or using the wrong dosage of drops^(5)^. Furthermore, older patients without support at home may face even greater challenges in correctly applying eye drops due to potential physical or cognitive impairments^(4)^. As a result, consistent adherence to the prescribed post-operative eye drop regimen can be unreliable in this demographic^(4)^.

Given the challenges associated with postoperative topical eye drop use, there is growing interest in establishing an effective dropless postoperative protocol^(6)^. In dropless cataract surgery, many surgeons globally have replaced topical antibiotics with an intracameral antibiotic solutions, which have shown favorable outcomes and reduced endophthalmitis rates, as reported by a Cochrane systematic review^(7)^. More recently, the dropless approach has been extended to include the administration of steroids and/or NSAIDs. A commonly adapted alternative is the intraoperative administration of a steroid depot to manage postoperative inflammation. Several techniques have been described, including sustained-release dexamethasone delivered via an intracanalicular insert or intraocular suspension, as well as intravitreal or subconjunctival injections of triamcinolone^(8^–^12)^. Among these, the subconjunctival injection method is becoming the most frequently utilized^(8^–^10^,^13^–^15)^. In a cohort study involving 69,382 patients who underwent cataract surgery between 2018 and 2021, some patients received subconjunctival triamcinolone injections at varying concentrations, while others were treated with topical steroids ± NSAIDs. The results indicated that the group receiving triamcinolone injections had a reduced likelihood of developing macular edema and iritis compared to those treated with topical steroids ± NSAIDs^(6)^. Moreover, other studies suggest that injections may offer greater cost-effectiveness compared to topical drops^(8^–^10^,^13^–^15)^. However, some evidence indicates that this dropless approach may be associated with a heightened risk of prolonged steroid response and elevated IOP postoperatively^(10)^. The overall benefits and risks of using perioperative subconjunctival steroid injections in dropless cataract surgery remain a topic of ongoing debate^(11^,^12^,^16)^.

In light of the ongoing debate and the absence of a comprehensive evaluation, this study aimed to evaluate the efficacy and safety of perioperative subconjunctival steroid injections in comparison with conventional topical steroids for managing postoperative inflammation after cataract surgery. To help bridge this important knowledge gap, we carried out a systematic review and meta-analysis, integrating data from multiple studies to assess the efficacy, safety, pharmacokinetics, tolerability, risks, potential benefits, and cost-effectiveness of perioperative subconjunctival steroid use.

## METHODS

### Eligibility criteria

Studies were included in our meta-analysis if they were randomized controlled trials (RCTs) or cohort studies, (2) if participants underwent cataract surgery and were assigned to receive a perioperative subconjunctival steroid injection as defined by the investigators, and if the studies evaluated (3) steroid response, (4) incidence of macular edema, (5) intraocular pressure, and (6) inflammation control. We excluded articles that only compared postoperative use of eye drops without addressing dropless surgery, those involving patients undergoing surgeries other than cataract surgery, and interventions that did not involve steroids. Additionally, abstracts, theses, case reports, opinion pieces, and correspondence articles were excluded. There was no minimum or maximum follow-up period required after surgery. When multiple IOP measurements were reported, we used data from the 1-week and 1-month postoperative time points in this study.

### Screening process

Two independent authors (AN, MA) initially screened the studies by title and abstract, followed by full-text review. In cases where eligibility was unclear, a third author (GG) reviewed the articles and made the final decision after discussion. All studies that met the predefined criteria were included.

### Search strategy and data extraction

We conducted a systematic search of PubMed, EMBASE, and Cochrane Central for observational trials and RCTs using the following search terms: “cataract”, “phaco”, “phacoemulsification”, “perioperative”, “perioperative steroid”, “dropless”, and “topical drops”. No restrictions were placed on publication date or language. Two authors (AN, MA) independently extracted relevant data from the selected studies using a standardized form, which included details such as authors, publication year, study design, sample size, and other pertinent information. Any disagreements were resolved by consensus following discussion with the senior author.

### Quality assessment

Two authors (OR, VE) conducted the quality assess ment using the Cochrane Collaboration’s RoB 2 tool to evaluate the risk of bias in RCTs. Studies were rated as having high, low, or unclear risk of bias across fives domains: selection, performance, detection, attrition, and reporting biases^(17)^. For non-randomized studies, the ROBINS-I tool was applied to assess risk of bias, rating studies as having serious, low, or moderate risk across seven domains: bias due to confounding, participant selection, intervention classification, deviations from intended interventions, missing data, outcome measurement, and selection of reported results^(18)^. Any disagreements were resolved by consensus following discussion with the senior author.

### Statistical analysis

Mean differences (MD) with 95% confidence intervals (CI) were applied for continuous outcomes, while risk differences (RD) with 95% CI were used to compare treatment effects for categorical outcomes. A p-value <0.05 was considered statistically significant for both MD and RD. The Cochran Q test and I^2^ statistics were employed to assess heterogeneity; significance was set at p<0.10 and I^2^ >40%^(19)^. When significant heterogeneity was detected, a leave-one-out analysis was performed to address the heterogeneity and assess the robustness of the findings. A random-effects model was utilized for all analyses.

## RESULTS

### Study selection and characteristics

**Figure 1:**
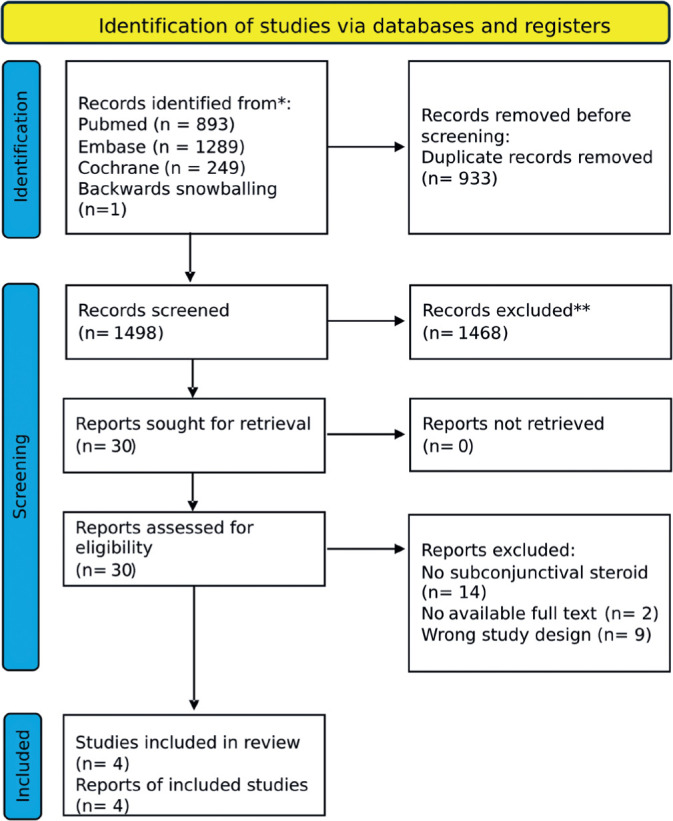
PRISMA flow diagram illustrating the study selection process

As shown in figure 1, the initial search identified 2,431 records. After duplicates and ineligible studies were removed, 30 articles remained and were carefully assessed against the inclusion criteria. Of these, two retrospective cohort studies, one non-randomized trial, and two RCTs were included, involving a total of 70,751 eyes. Among the patients, 12,319 (17.4%) received perioperative subconjunctival steroid injections, while 58,432 (82.6%) were treated with prednisolone acetate combined with NSAIDs. Details of the study characteristics are provided in table 1.

**Table 1:** Design and characteristics of the studies included in this meta-analysis

	Type of analysis	No. of patients/eyes	Follow-up period	Population	Intervention	Control
**Shorstein et al., 2024** ^(14)^	RC	I: 11.893 C: 57939	January 1, 2018, to December 31, 2021	Patients aged ≥18 years undergoing phacoemulsification cataract surgery	Subconjuntival TA 10 mg/ml or 40 mg/ml with low dose (1.0–3.0 mg) or high dose (3.1–5.0 mg)	Topical PA alone or PA combined with NSAIDs
**Wu et al., 2023** ^(10)^	RC	I:150 C: 218	January 1, 2020, to September 30, 2021	Patients undergoing cataract surgery	Subconjunctival depot of triamcinolone 12–20 mg	Topical prednisolone
**Lindholm et al., 2020** ^(9)^	Non-RCT	I: 51 C: 50	April 2017 to October 2017	Patients aged 60–90 years eligible for cataract surgery	Single subconjunctival 0.5-ml injection of TA suspension (40 mg/ml)	Dexamethasone 1 mg/ml eye drops
**Dieleman, et al., 2011** ^(8)^	RCT	I: 200 C: 200	February 2007 to June 2009	White patients scheduled for routine phacoemulsification at Rotterdam Eye Hospital	Perioperative subconjunctival injection of betamethasone acetate 5.7 mg/mL	Dexamethasone 0.1% eye drops
**Merkoudis et al., 2014** ^(13)^	RCT	I: 25 C: 25	2010–2011	Patients aged 60–90 years scheduled for routine phacoemulsification at Uppsala University Hospital	Single subconjunctival injection of 20 mg methylprednisolone acetate	Dexamethasone 1 mg/ml eye drops

### Pooled analysis of all studies

**Figure 2:**
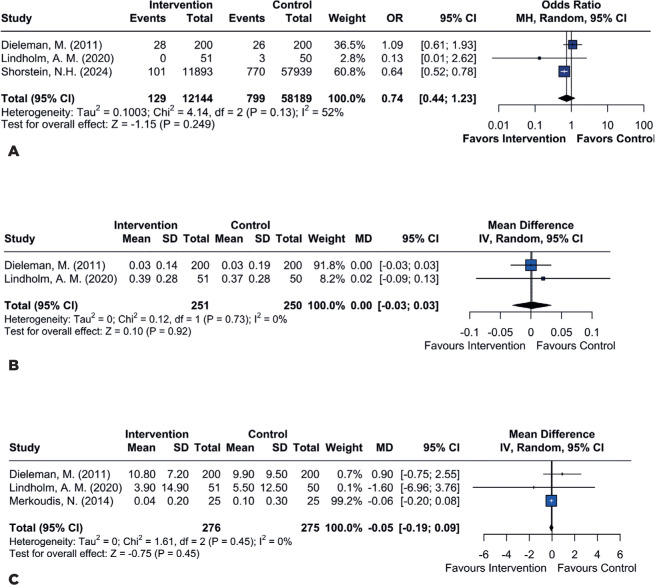
Comparison between the perioperative subconjunctival steroid group and the control group for the following outcomes: **(A)** no significant difference in macular edema; **(B)** no significant difference in visual acuity; **(C)** no significant difference in laser flare count.

**Figure 3:**
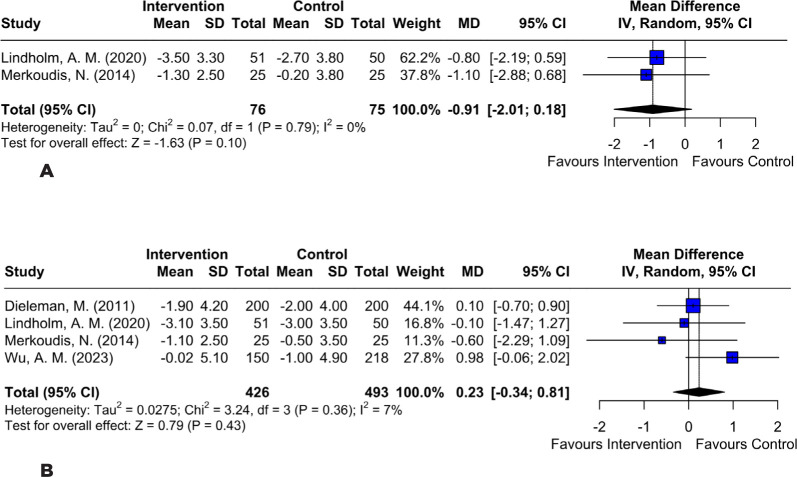
Comparison between the perioperative subconjunctival steroid group and the control group for the following outcomes: **(A)** no significant difference in intraocular pressure at 7 days; **(B)** no significant difference in intraocular pressure at 1 month.

No statistically significant difference was observed in the incidence of macular edema between the treat ment and control groups (odds ratio [OR] 0.74; 95% CI 0.44–1.23; p=0.249; *I*²=52%; Figure 2A). Similarly, there were no significant differences in visual acuity (MD 0.00; 95% CI −0.03–0.03; p=0.73; *I*²=0%; Figure 2B), laser flare count (MD −0.05; 95% CI −0.19–0.09; p=0.45; *I*²=0%; Figure 2C), IOP at 7 days postoperative (MD −0.91; 95% CI −2.01–0.18; p=0.10; *I*²=0%; Figure 3A), or IOP at 1 month postoperative (MD 0.23; 95% CI −0.34–0.81; p=0.43; *I*²=7%; Figure 3B).

**Figure 4:**
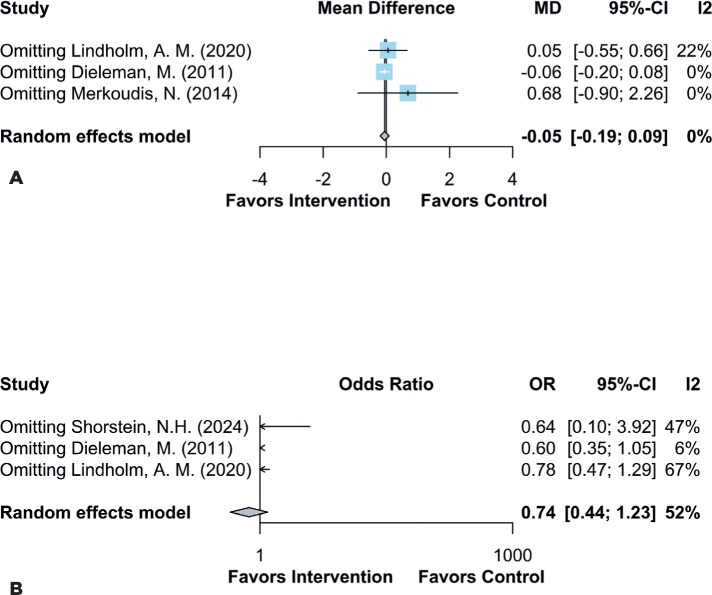
Leave-one-out analysis for these outcomes: **(A)** no significant difference in laser flare count; **(B)** no significant difference in macular edema (CI, confidence interval; SD, standard deviation).

In instances where high heterogeneity was detected, a leave-one-out analysis was performed to address this heterogeneity and assess the robustness of the results. This analysis confirmed no statistically significant difference in laser flare count (MD −0.05; 95% CI -.0.19–0.09; p=0.249; *I*²=0%; Figure 4) or macular edema (OR 0.74; 95% CI 0.44–1.23; *I*²=52%; Figure 4) compared with the control group.

### Quality assessment

**Figure 5:**
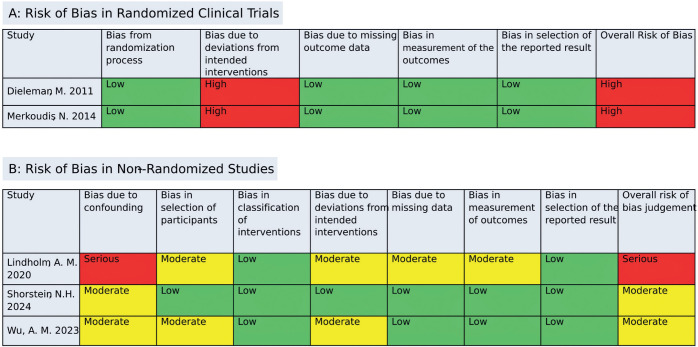
Risk of bias assessment for the included studies

Using the RoB 2 tool, two studies were found to have a serious risk of bias due to deviations from the intended interventions^(13^,^20)^. According to the ROBINS-I assessment, one study was classified as having a serious risk of bias from confounding and a moderate risk of bias in four additional domains^(9)^. The other two studies were rated as having a moderate risk of bias: one related to confounding and the other involving confounding, participant selection, and deviations from intended interventions^(10^,^14)^. A detailed evaluation of each study across all RoB 2 and ROBINS-I domains is presented in figure 5.

## DISCUSSION

In this meta-analysis, we evaluated the efficacy and safety of subconjunctival steroid injections versus traditional topical steroids for managing postoperative inflammation after cataract surgery. Our review of five studies—Shorstein et al., Lindholm et al., Dieleman et al., Wu et al., and Merkoudis et al.—showed no statistically significant differences in important outcomes such as macular edema, visual acuity, laser flare counts, and IOP^(8^-^10^,^13^,^14)^.

The absence of a statistically significant difference in macular edema (OR 0.74; 95% CI 0.44–1.23; p=0.249; I²=52%) aligns with Lindholm et al.’s findings, which demonstrated effective prevention of macular edema with both subconjunctival and topical treatments^(9)^. Likewise, none of the studies, including Merkoudis et al., reported significant differences in final visual acuity between the two treatment methods (MD 0.00; 95% CI −0.03–0.03; p=0.73; I²=0%)^(13)^.

In assessing inflammation using laser flare counts, the lack of statistically significant differences (MD −0.05; 95% CI −0.19–0.09; p=0.45; I²=0%) supports the findings of Dieleman et al., indicating similar efficacy in controlling postoperative inflammatory between the two treatments^(20)^.

Regarding IOP, a key concern noted by Wu et al., our analysis found no significant difference between subconjunctival and topical steroid treatments at both 7 days (MD −0.91; 95% CI −2.01–0.18; p=0.10; I²=0%) and 1 month after surgery (MD 0.23; 95% CI −0.34–0.81; p=0.43; I²=7%)^(10)^. Although Shorstein et al. reported possible increases in IOP with depot steroids, our combined analysis did not find a significant difference, suggesting that, with appropriate monitoring, both approaches are similarly safe in terms of risk for ocular hypertension^(14)^.

These results indicate a strong consistency among the reviewed studies, with no single method showing clear superiority. The findings challenge the assumption that subconjunctival injections always provide better clinical outcomes because of prolonged drug release, as noted by Merkoudis et al.^(13)^ Rather, the decision between delivery methods should prioritize individual patient characteristics, potential issues with adherence, and overall convenience of treatment instead of focusing solely on efficacy.

Adherence to topical eye drop regimens remains a significant challenge in clinical settings. Correct eye drop administration depends on visual acuity, manual dexterity, and comprehension of treatment instructions^(21)^. A multicenter study in Latin America involving patients with chronic eye conditions found that fewer than 55% had adequate adherence to topical treatments^(22)^. These challenges are particularly relevant in managing postoperative cataract patients, who often require multiple medications over days or weeks. The dropless approach, using perioperative subconjunctival steroid injections, may overcome these difficulties by removing the need for patient-applied drops, thereby improving adherence and standardizing postoperative care. In public health systems such as the *Sistema Único de* *Saúde* (SUS), where follow-up resources might be limited, this approach could promote greater treatment equity and better surgical outcomes across varied patient groups.

This meta-analysis has several limitations that should be considered when interpreting the findings. First, although all included studies used depot steroids, there were notable differences in the methods regarding the specific drugs and dosages administered. Shorstein et al., Lindholm et al., Dieleman et al., Wu et al., and Merkoudis et al. used various types and concentrations of steroids, which introduced heterogeneity and may have affected the comparability of results^(8^-^10^,^13^,^14)^. Additionally, the limited number of studies on this subject restricts the generalizability of the conclusions to a wider population. Another limitation is the inconsistency in the criteria used to define and measure outcomes such as macular edema and inflammation, as these were not standardized across the studies. It is also important to note that none of the studies were blinded, which could lead to bias in outcome assessments. Most of the included studies were found to have a moderate to high risk of bias, particularly due to confounding factors, lack of blinding, and deviations from intended interventions. These methodological limitations may affect the internal validity of individual studies and decrease the overall confidence in the combined results. Therefore, the findings of this meta-analysis should be interpreted carefully. Additional RCTs are needed to further evaluate the clinical equivalence of subconjunctival and topical steroid treatments in cataract surgery.

In conclusion, this meta-analysis demonstrates that subconjunctival steroid injections and conventional topical steroids are similarly effective in controlling postoperative inflammation after cataract surgery, with no statistically significant differences observed in key outcomes.

## Data availability statement:

The datasets generated and/or analyzed during the current study will be made available at time of publication of the article.
